# Establishment of a novel human nervous system tissue bank: University of Debrecen tissue bank—introduction and retrospective data analysis

**DOI:** 10.1007/s10561-026-10249-9

**Published:** 2026-09-02

**Authors:** Viktor Bencs, Tibor Hortobágyi, Attila Csaba Nagy, Márk Kozák, Gábor Méhes, László Oláh, László Csiba

**Affiliations:** 1 Department of Neurology, Faculty of Medicine, University of Debrecen, Debrecen, Hungary; 2 Institute of Neuropathology, University Hospital Zürich, Zurich, Switzerland; 3 Department of Epidemiology, Faculty of Medicine, University of Debrecen, Debrecen, Hungary; 4 Department of Infectology, Faculty of Medicine, University of Debrecen, Debrecen, Hungary; 5 Department of Pathology, Faculty of Medicine, University of Debrecen, Debrecen, Hungary

**Keywords:** Brain bank, Formalin-fixed paraffin-embedded (FFPE) tissue, Autopsy-based research, Neurodegenerative disorders, Neuropathological database

## Abstract

Human brain tissues obtained from autopsies, as well as muscle and nerve samples derived from biopsies, constitute indispensable resources for translational research investigating the pathomechanisms of neuropsychiatric disorders. In this study, we introduce the Nervous System Tissue Bank of University of Debrecen, which contains formalin-preserved and formalin-fixed paraffin-embedded (FFPE) brain samples, as well as frozen nerve and muscle biopsy specimens. Database was established containing clinical and pathological parameters. For autopsy cases, the following variables were recorded: sex, age, number of hospitalization days, admission diagnosis, comorbidities, cause of death, post-mortem interval, general autopsy findings, and macro- and microscopic brain pathology diagnoses. For biopsy samples, we recorded: sex, age, biopsy site, and microscopic and molecular genetic diagnoses. Based on the recorded parameters and their temporal changes, retrospective statistical analyses were performed. Formalin-fixed brain samples and FFPE blocks were available from 2425 deceased individuals, and nerve/muscle samples from 257 patients. The brain samples originate from the period between 1990 and 2012. The most frequent admission diagnoses included ischemic stroke (n = 1070), hemorrhagic stroke (n = 462), subarachnoid hemorrhage (n = 102), epilepsy (n = 103), brain metastasis (n = 70), primary brain tumor (n = 60), parkinsonism (n = 28), dementia (n = 21), and amyotrophic lateral sclerosis (n = 21). Prevalence of hypertension (67.6%) and diabetes mellitus (20.1%) showed a significant increase over time (*p < *0.001), whereas the prevalence of malignancies, dementia, parkinsonism, and psychiatric disorders remained stable. Our tissue bank represents the largest nervous system collection in Hungary, providing well-characterized brain, muscle, and nerve samples linked to nearly three decades of clinical data. Samples are made available to researchers.

## Introduction

Pathophysiological mechanisms of numerous neurological and psychiatric disorders remain poorly understood. These disorders impose a substantial financial and social burden on society; therefore, development of early and effective diagnostic tools and appropriate therapies is critical for the future. (Feigin et al. 2020) To achieve this, basic research is needed to clarify the underlying pathophysiological mechanisms of these conditions. Cell cultures, organ-mimicking cell systems, animal models, and animal tissues can all be employed to investigate cellular signaling pathways and potential drug targets; however, the disease-specific molecular microenvironment is most relevant in human tissue samples. Consequently, examination of human tissue samples would be ideal; however, their availability to researchers is limited due to legal constraints, as well as challenges and costs associated with sample collection and storage. Human neural tissue can be obtained through two primary methods: biopsy or autopsy. Brain biopsy is an invasive and high-risk surgical procedure, typically reserved as a last resort in the diagnostic workup of conditions where a definitive diagnosis cannot be achieved by other means. Brain biopsies are not performed for purely scientific purposes; therefore, autopsy remains the main source of human brain tissue for research. In contrast, sampling of the peripheral nervous system—such as muscle and nerve biopsies—is more commonly performed in vivo, particularly in the context of neuromuscular disorders. Consequently, biopsies represent the primary source of peripheral nervous system tissue samples. Regarding to sample preservation, two main methods are available: cryopreservation in fresh state and formalin fixation. Cryopreservation is a time- and resource-intensive technique that requires careful handling; however, it provides the most suitable conditions for conducting biochemical, genetic and proteomic analyses. Protocols for cryopreservation vary across institutions. Autopsy and tissue sampling must be performed as soon as possible after death, preferably no later than 24 h post-mortem. The excised tissue should be immediately placed on dry ice at 4 °C, then transferred to a pre-cooled aluminum tray and snap-frozen in liquid nitrogen at − 120 °C, followed by storage at − 80 °C. In contrast, formalin fixation is a far simpler technique with less stringent post-mortem interval requirements. After brain removal, the tissue is immersed in 10% phosphate-buffered formalin solution, which is replaced with fresh fixative after 24 h. The brain is then stored for three weeks in a cold, dark room. Subsequently, the tissue can either remain stored in formalin or undergo further processing, including paraffin embedding and histological preparation. (Poloni et al. 2020) Most brain banks routinely preserve both cryopreserved and formalin-fixed samples from each donor to ensure comprehensive investigational possibilities. Although inclusion criteria, autopsy procedures, and tissue processing protocols may vary between brain banks, there have been several initiatives to establish brain bank networks aimed at methodological standardization and improved accessibility of high-quality samples for research purposes (e.g., BrainNet Europe, UK Brain Bank Network). (Shepherd et al. 2019) It is a widely accepted fact that frozen tissue samples are the most suitable for molecular genetic analyses (such as DNA, RNA, exome, and genome sequencing). (Ferrer et al. 2007) In recent years, however, numerous studies have investigated and confirmed that formalin fixed paraffin-embedded (FFPE) samples may also be suitable for this purpose when appropriate analytical techniques are employed. (Funabashi et al. 2012; Wang et al. 2013) Vitošević et al. revealed, that short amplicons of DNA isolated from long-term FFPE tissues can be efficiently analysed and used for investigational and diagnostic applications. However, these DNA samples do not seem to be suitable for analysis of longer DNA amplicons. FFPE brain tissues are stable for histological analysis after long period of storage. (Vitošević et al. 2021) Several studies have reported that during histological analysis of FFPE blocks, using routine staining techniques, no morphological differences can be observed between long-stored and more recently archived samples. Vitošević et al. demonstrated this in samples stored for 30 years, while Bass et al. reported similar findings in samples stored for up to 48 years. (Bass et al. 2014; Vitošević et al. 2021).

Beyond sample provision, autopsies also serve several other significant roles: it is a diagnostic procedure that provides critical information about the events occurring between the last clinical presentation and death. It aids in establishing the definitive diagnosis and offers an opportunity to verify whether appropriate medical treatment was administered. Despite these essential roles, the global rate of traditional autopsies has been steadily declining. Whereas autopsy rates in most European countries and the United States were around 60% half a century ago, these have now fallen below 5%. (Goldman 2018) As a result, numerous comorbidities (e.g., malignancies, systemic diseases), clinically unrecognized complications (e.g., infections, thromboembolic events), and adverse effects of therapy may remain undetected. The most pressing issue, however, is the diminishing opportunities to collect, process, and preserve new human tissue samples for research. The value of formalin-fixed tissue samples accumulated over the years in pathology departments archives has become increasingly significant. This study aims to present the development of a newly established human tissue bank comprising samples from both central and peripheral nervous systems: the University of Debrecen Nervous System Tissue Bank in Debrecen, Hungary.

## Materials and methods

### Central nervous system tissues

At the Department of Neurology, University of Debrecen, autopsies of patients who had died with neurological and psychiatric disorders were performed at the Department of Pathology between 1990 and 2012. For these cases, we possess comprehensive clinical, laboratory, general autopsy, and neuropathological data. The brains have been preserved either as whole specimens fixed in formalin or as paraffin-embedded sections prepared from well-documented, dissected regions. During autopsy at the Pathology Department, the brain was removed in its entirety, weighed, and subjected to external macroscopic examination which includes assessment of the leptomeninges, gyral pattern, vessels of the Circle of Willis and its major branches, and any focal abnormalities observed in the brainstem, cerebellum, and cerebral hemispheres. After this step the brain was immersed in 10% phosphate-buffered formalin and transferred to the Laboratory of Neuropathology where neuropathologist conducted the further examinaton. The formalin solution is replaced entirely the day after immersion and subsequently on a weekly basis. After three weeks of fixation, tissue processing can begin. Using the Spielmeyer technique (Sejda et al. 2022), the cerebellum and brainstem are separated, followed by the preparation of 0.75 cm thick coronal sections of cerebral hemispheres, cerebellar hemispheres and 2 mm thin slices of the brainstem. Subsequently, a macroscopic examination of the brain slices is performed, followed by the identification of specific regions to be dissected from the slices and embedded in paraffin for histological analysis by standard procedures.(Latimer et al. 2023) The brain regions selected for histological examination depended on the time of autopsy. Between 1989 and 2009, sections were primarily obtained from the clearly pathological areas, and from non-affected areas for comparison, and, and only hematoxylin and eosin (H&E) staining was used for the standard histological analysis. From 2010, tissue processing became standardized and more comprehensive. In each case, tissue blocks were taken from the following regions: middle frontal gyrus, superior and middle temporal gyri, anterior hippocampal and parahippocampal gyri, superior margin of the intraparietal sulcus, midbrain, superior frontal gyrus, calcarine sulcus, striatum, amygdala, thalamus, pons, medulla oblongata, cerebellum, deep white matter of the frontal and occipital lobes, posterior hippocampus, and clearly pathological area, according to internationally accepted sampling protocols.(Klioueva et al. 2015) During this period, in addition to the routine hematoxylin and eosin staining, beta-amyloid, alpha-synuclein, tau, TDP-43 immunohistochemistry and and Luxol fast blue staining were performed in relevant regions. As a result, neurodegenerative pathologies were investigated even in cases where dementia was not the primary clinical diagnosis at the time of admission. In accordance with the procedures described above, the brains were preserved as formalin-fixed coronal slices, and paraffin-embedded histological blocks were also prepared from these sections. The tissue samples are stored in a cold, dark room and are available for future research use. The human tissue samples have been available for several years; however, previously no centralized registry existed that included the clinical, pathological, and neuropathological data associated with the cases. Therefore, our aim was to establish a unified database from the stored brains, which could be made accessible to international research groups investigating the pathomechanisms of neuropsychiatric disorders using basic research methodologies on formalin-fixed samples. Furthermore, as we have collected nearly three decades’ worth of clinical and pathological data, we conducted a retrospective analysis of various parameters of individuals who died in our clinic. As the first step of our work, we checked the quality and quantity of the stored tissue samples. Subsequently, we obtained the clinical discharge summaries, general autopsy reports, and neuropathological examination reports corresponding to the tissue samples. From the documentation, we extracted the following parameters for inclusion in the database: age, sex, number of hospital days until death, primary diagnosis requiring hospital admission and related clinical information, previously known underlying conditions (with special attention to hypertension, diabetes mellitus, malignancies, psychiatric disorders, parkinsonism and other movement disorders, and dementia), cause of death as determined by the clinician, neuropathological findings (both macroscopic and microscopic abnormalities), general autopsy data (post-mortem interval, tumors or other significant diseases discovered during autopsy, and cause of death as determined by the pathologist).

### Retrospective analysis of pathological data

As outlined earlier, autopsy continues to serve as the gold standard for assessing the quality of inpatient medical care. During sample processing, we reviewed over 25 years of clinical and pathological data; therefore, our study places special focus on the insights gained from this extensive dataset. We investigated temporal trends in the number of autopsies performed, as well as changes in age and sex distribution, length of hospital stay, and the proportion of admission diagnoses over time. STATA software was used for statistical analysis.

### Peripheral nervous system tissues

Tissues from peripheral nervous system includes muscle and nerve samples. These samples were obtained for diagnostic purposes from living individuals during surgical procedures. Following removal, the samples were immediately transferred to the Department of Pathology, where the necessary processing was performed for histological examination, electron microscopy, or, if needed, molecular genetic analysis. The remaining tissue was promptly snap-frozen in liquid nitrogen. We have archived muscle and nerve biopsy samples stored in liquid nitrogen from the Department of Neurology collected between 2014 and 2018. For these samples, the following parameters were recorded in our database: sex, age at the time of biopsy, biopsy site, clinical indication for biopsy (clinical question), availability of routine histological, electron microscopic, or molecular pathological reports, and definitive diagnosis.

## Results

### Central nervous system tissues

We have tissue samples from a total of 2425 individuals who died from neuropsychiatric disorders. These include formalin-fixed specimens and paraffin-embedded tissues. In majority of cases, whole brains sectioned in the coronal plane and preserved in formalin are available, along with paraffin blocks dissected from well-documented brain regions of these specimens.

### *Changes in the number of *stored samples* over the years*

**Fig. 1:**
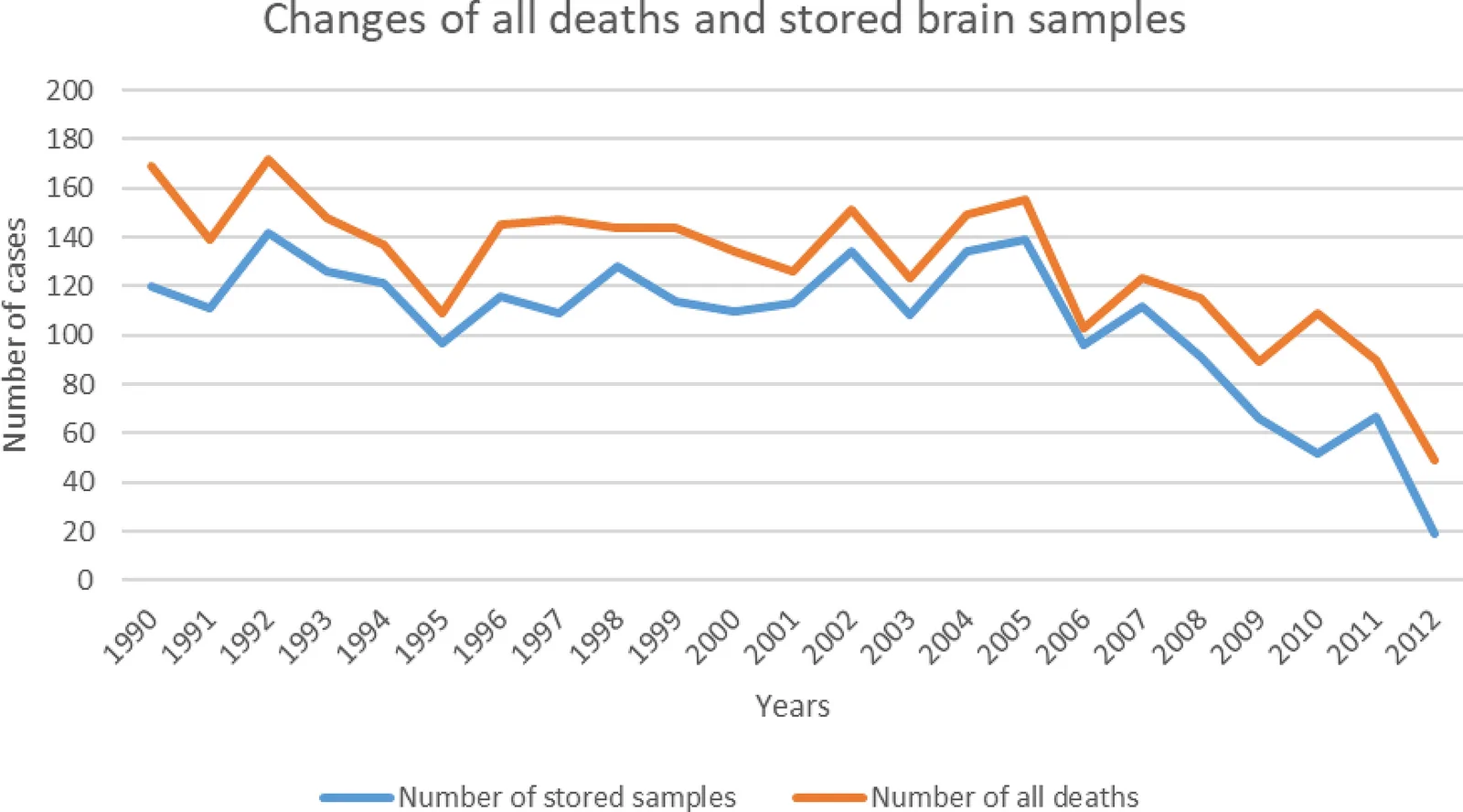
The diagram shows the yearly changes between 1990 and 2012 in the total number of deaths and the number of stored brain samples. It compares the trends of these two variables over time, highlighting that both follow a generally similar pattern but decline markedly in the later years

Brain tissue samples are available from the period between 1990 and 2012. The annual variation in stored sample numbers and all deaths in our institution is illustrated in Fig. 1. The number of annual autopsies remained consistently above 95 dissections/year throughout the 1990s and early 2000s. However, a marked decline began in 2007.

### Sex distribution over the years

**Fig. 2:**
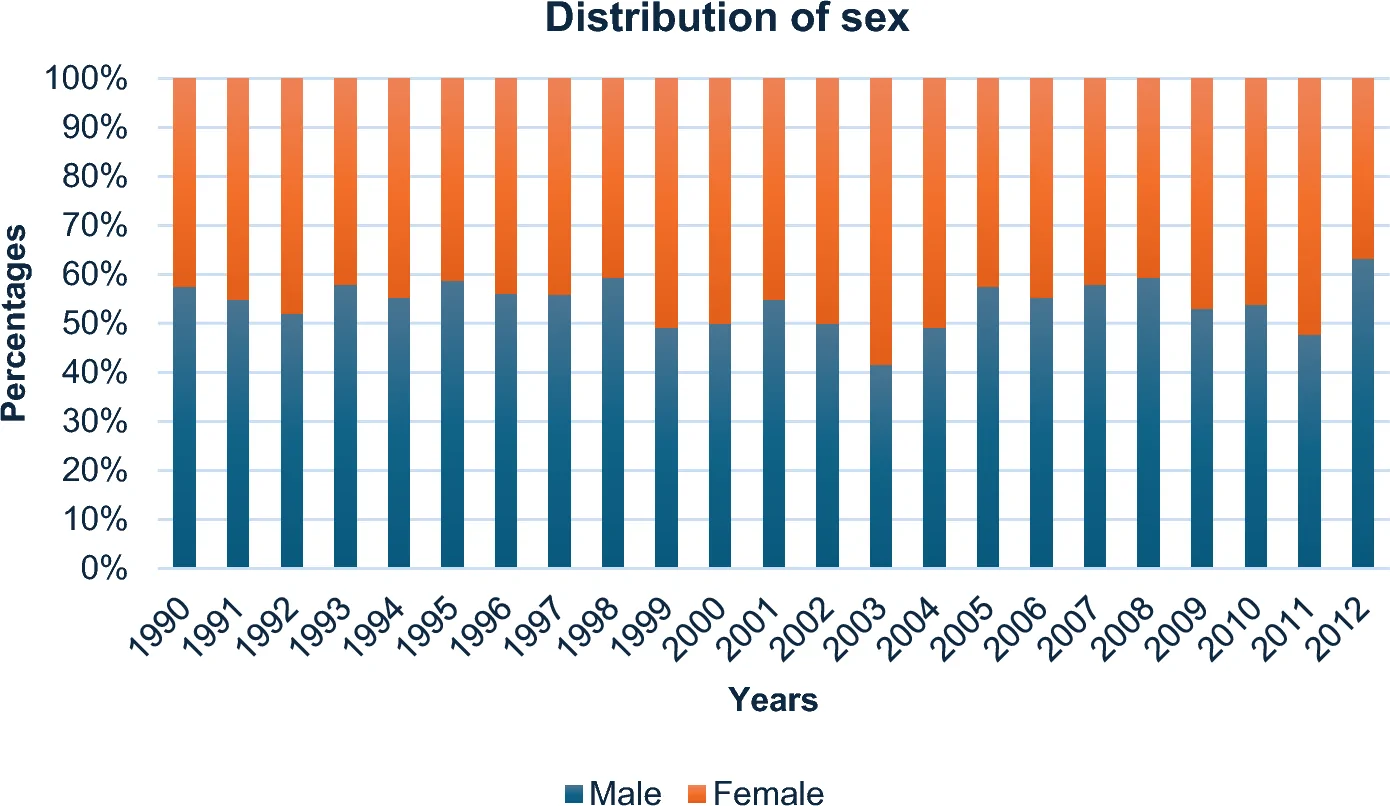
Annual sex distribution. The vertical axis indicates the sex distribution of the autopsied brain samples. Male cases are represented in blue, while female cases are shown in orange. The horizontal axis shows the years

Brain samples from 1313 (54.14%) male and 1112 (45.86%) female deceased patients were available. Figure 2 illustrates the changes in sex distribution over the years. Except for the years 1999, 2003, 2004 and 2011 male individuals consistently outnumbered females among the deceased in all other years. In 2000 and 2002 the distribution was equal.

### Changes of ages at death

**Fig. 3:**
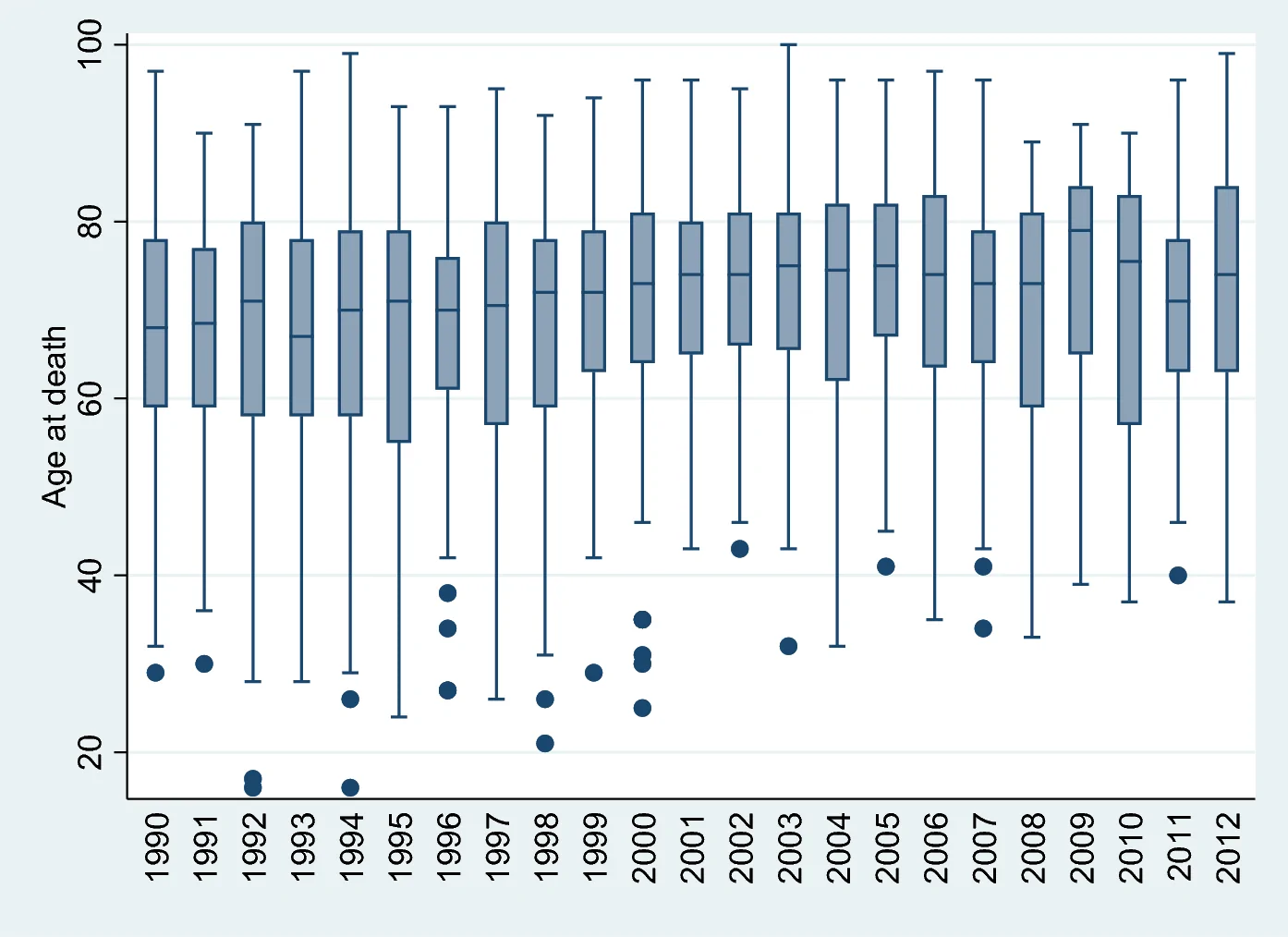
Boxplot chart shows the age at death per years. The vertical axis represents the age at death, while the horizontal axis corresponds to the years. The figure displays the median values, 25th and 75th percentiles, standard deviation, and outlier values for each year

Based on Shapiro–Wilk test, the age at death did not follow a normal distribution in the study population. Median value was 72 [61–80] years, minimum value was 16 years, maximum value was 100 years. Figure 3 presents the age at death stratified by year using a boxplot. Applying a linear regression model, the age at death was found to increase significantly over time (*p*<0.0001).

### Changes of days in hospital to death

**Fig. 4:**
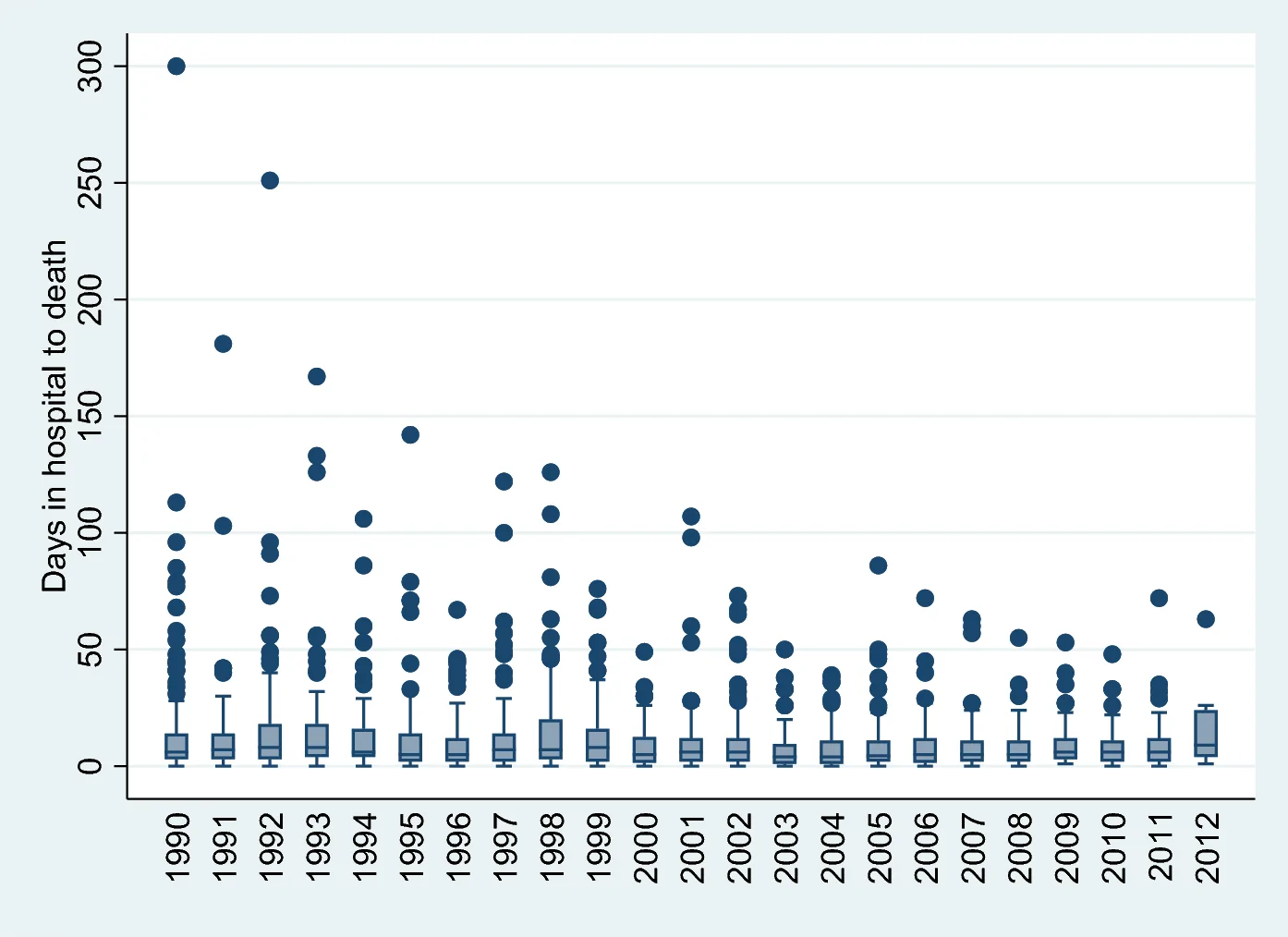
Boxplot chart shows the days in hospital to death per years. The vertical axis represents the days spent in hospital to death, while the horizontal axis corresponds to the years. The figure displays the median values, 25th and 75th percentiles, standard deviation, and outlier values for each year

Based on Shapiro–Wilk test, days in hospital to death did not follow a normal distribution in the study population. Median value was 6 (2–13 days), minimum value was 0 days, maximum value was 300 days. Figure 4 presents the days in hospital to death stratified by year using a boxplot. The figure shows considerable variability in the number of days spent in the hospital. While most patients stayed only a few days before death (median: 4–9 days), there were cases with hospital stays lasting several hundred days, as well as instances of death occurring on the day of admission. Applying a linear regression model, the days in hospital to death was found to decrease significantly over time (*p*<0.0001).

### Main reason of referral to hospital

**Fig. 5:**
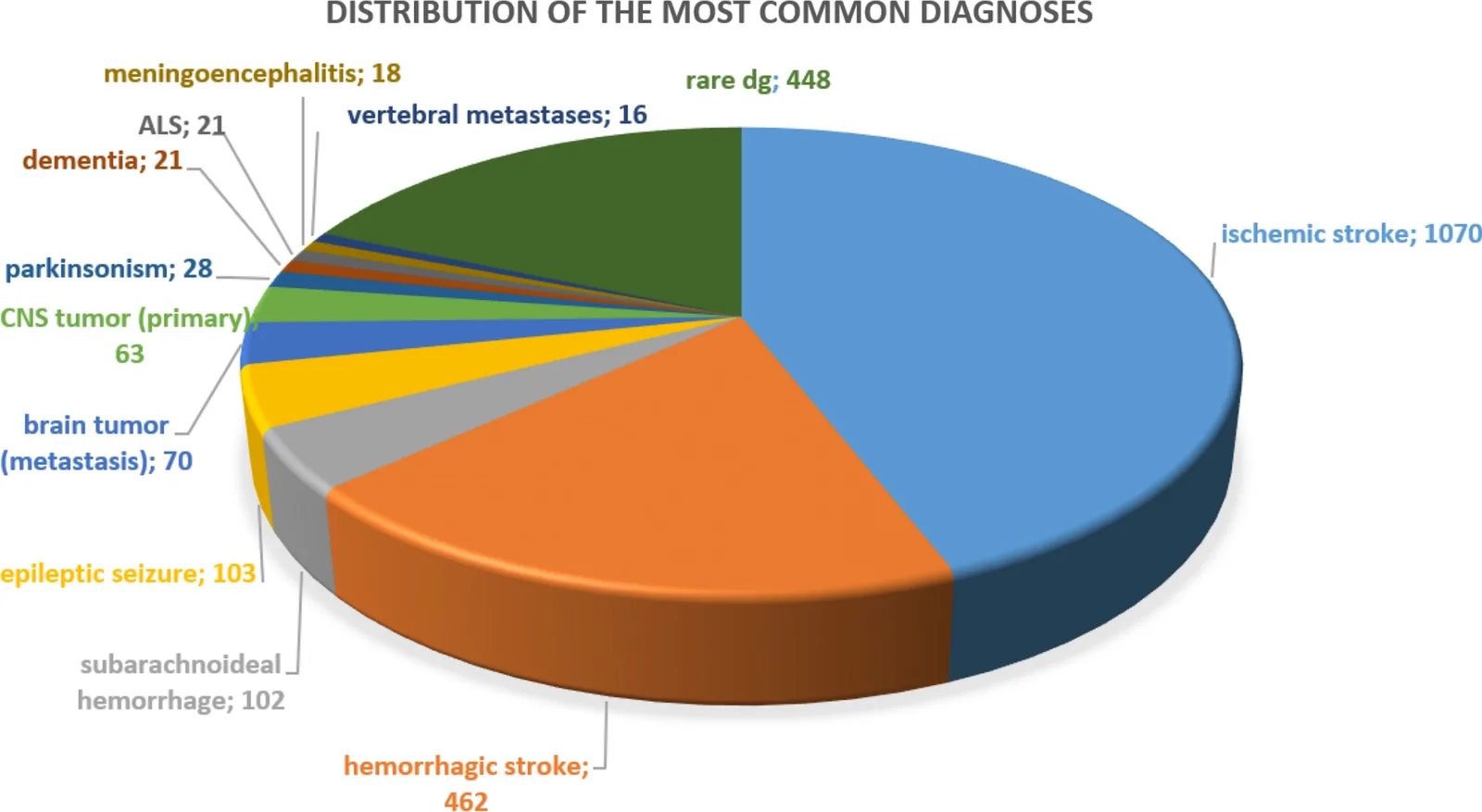
Pie chart with the distribution of the most common diagnoses

This parameter shows the main diagnosis of last referral of hospital which resulted in death. The referral diagnoses were ischemic stroke (1070 cases), hemorrhagic stroke (462 cases), epileptic seizure (103 cases), subarachnoideal hemorrhage (102 cases), metastatic brain tumor (70 cases), CNS tumor (primary) (63 cases), parkinsonism (28 cases), dementia (21 cases), ALS (21 cases), meningoencephalitis (18 cases), vertebral metastases (16 cases). Distribution of most common diagnoses is shown in Fig. 5. There are brain samples from several other diagnoses, including meningeal carcinomatosis, brain tumors with unknown origin, paranoid psychosis, unknown progressive neurological diseases, encephalitis, subdural hematoma, encephalopathies, delirium, acute myocardial infarction, bipolar depression, paraneoplastic syndrome, TIA, polyneuropathy, brain hypoxic lesion, schizophrenia, central pontine myelinolysis, unconsciousness without known reason, brain abscess, sinus thrombosis, lower back pain, depression, spinal cord compression, malignant neuroleptic syndrome, hypertensive encephalopathy, pneumonia, head trauma, myasthenia gravis, multiple sclerosis, sepsis, funicular myelosis, contusion brain hemorrhage, arteriosclerotic dementia, polyganglioradiculoneuritis, Ekbom syndrome, Wernicke encephalopathy, Duchenne muscular dystrophy, Korsakow psychosis, respectively.

### Comorbidites

#### Hypertension

**Fig. 6:**
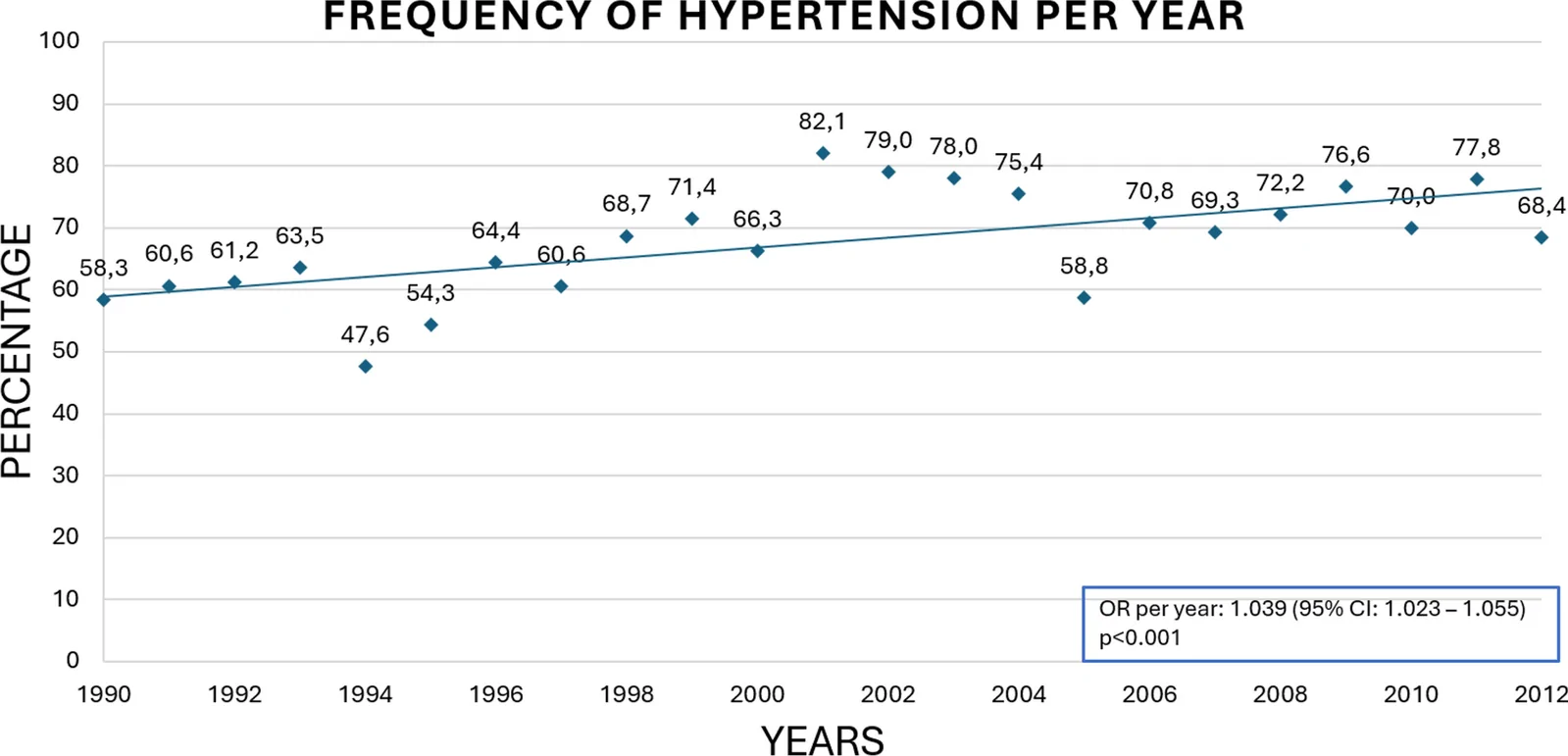
illustrates the annual prevalence of hypertension, expressed as a percentage, across the study period from 1990 to 2012. Each data point represents the proportion of individuals diagnosed with hypertension in a given year, while the fitted linear trend line depicts the overall temporal pattern

Hypertension was present in 67.6% of the deceased individuals. The annual variation in the percentage distribution of hypertension is illustrated in Fig. 6. Using a logistic regression model, the prevalence of hypertension was found to have increased significantly over time (*p*<0.001; OR: 1.0388).

#### Diabetes mellitus

**Fig. 7:**
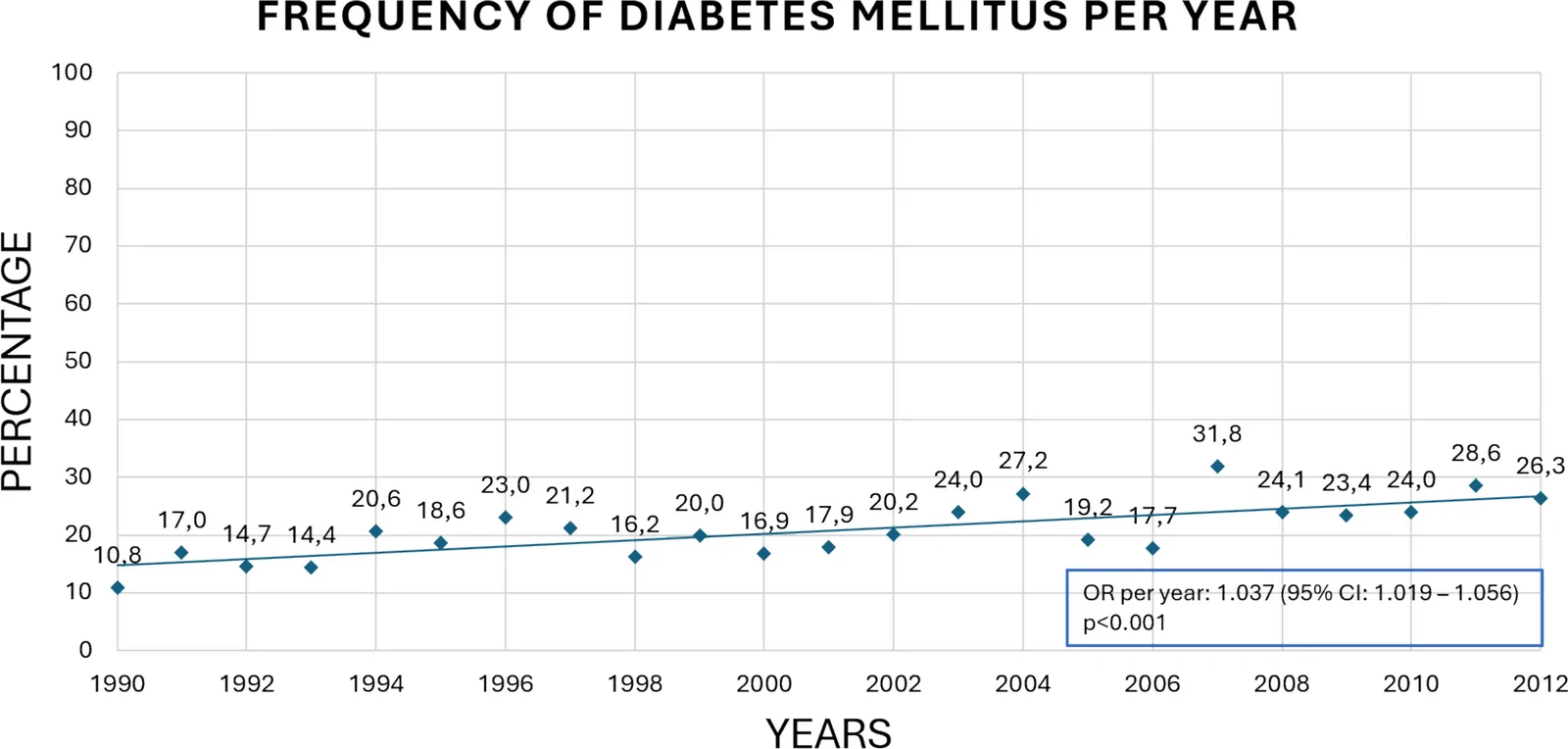
illustrates the annual prevalence of diabetes mellitus, expressed as a percentage, across the study period from 1990 to 2012. Each data point represents the proportion of individuals diagnosed with diabetes mellitus in a given year, while the fitted linear trend line depicts the overall temporal pattern

Diabetes mellitus was present in 20.1% of the deceased individuals. The annual variation in the percentage distribution of diabetes mellitus is illustrated in Fig. 7. Using a logistic regression model, the prevalence of diabetes was found to have increased significantly over time (*p*<0.001; OR: 1.037).

#### Cancer

**Fig. 8:**
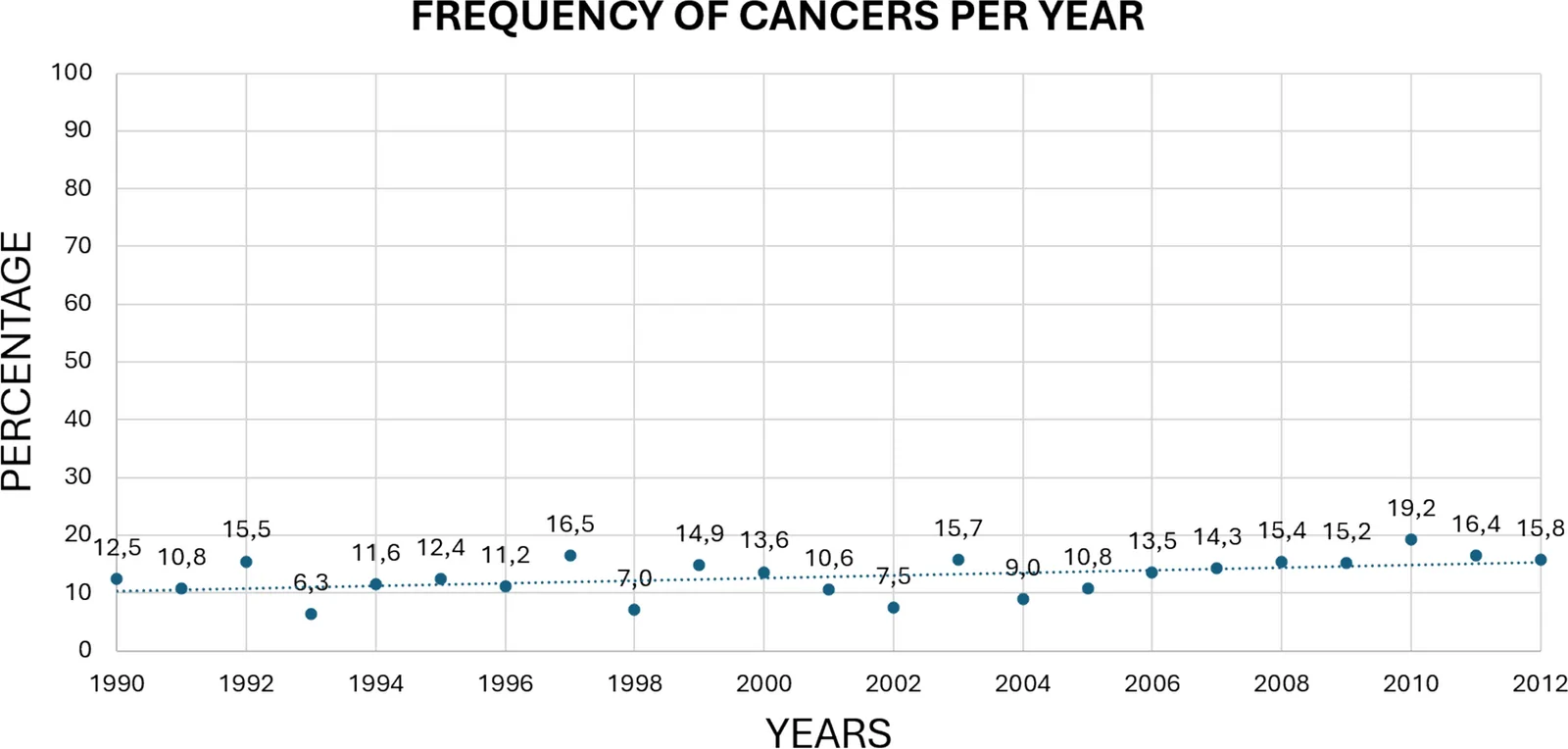
illustrates the annual prevalence of cancers, expressed as a percentage, across the study period from 1990 to 2012. Each data point represents the proportion of individuals diagnosed with cancer in a given year, while the fitted linear trend line depicts the overall temporal pattern

Cancer was diagnosed in 12.3% of the deceased individuals. The annual variation in the percentage distribution of cancer is illustrated in Fig. 8. Using a logistic regression model, no significant change was observed in the prevalence of tumors over the years.

The distribution of the tumor type for each year is summarized in Table 1. The “Brain” category includes malignant and benign intracranial tumors as well. “Hematologic” category includes lymphomas and leukemias. “Non-colorectal gastrointestinal” category includes oesophagus, gastric, liver, gall bladder, pancreas cancer. “Other” category includes phaeochromocytoma, orbita, skeletal, thyroid gland, larynx, oral, and spinal cord cancers. “Skin” category includes malignant melanoma and non-melanomatous skin cancers.

**Table 1:** Annual distribution of tumor spectrum

Years	brain	breast	colorectal	hematologic	lung	non-colorectal gastrointestinal	other	skin	urogenital
1990	2	3	2	0	5	1	0	0	2
1991	1	1	1	0	2	1	3	1	1
1992	5	2	3	0	4	1	2	1	3
1993	2	0	1	0	1	0	0	1	3
1994	8	0	0	0	4	0	0	0	2
1995	2	0	2	0	2	2	2	1	1
1996	1	0	1	0	4	2	2	0	2
1997	5	2	3	0	5	0	0	2	1
1998	1	0	0	0	2	2	0	1	2
1999	4	1	2	1	4	0	1	2	1
2000	2	2	1	2	1	0	3	1	2
2001	0	1	2	0	5	1	0	1	2
2002	2	0	2	0	2	0	2	0	2
2003	4	2	3	0	1	0	0	2	4
2004	1	1	1	1	4	1	0	1	0
2005	5	1	2	0	1	0	0	1	3
2006	0	0	3	1	2	0	1	2	2
2007	2	3	4	1	1	2	2	0	1
2008	2	1	3	0	1	0	2	2	2
2009	1	0	0	2	1	1	2	2	0
2010	0	1	1	0	3	2	0	1	1
2011	0	4	1	0	0	2	1	0	2
2012	1	0	0	2	0	0	0	0	0
SUM	51	25	38	10	55	18	23	22	39

#### Dementia

**Fig. 9:**
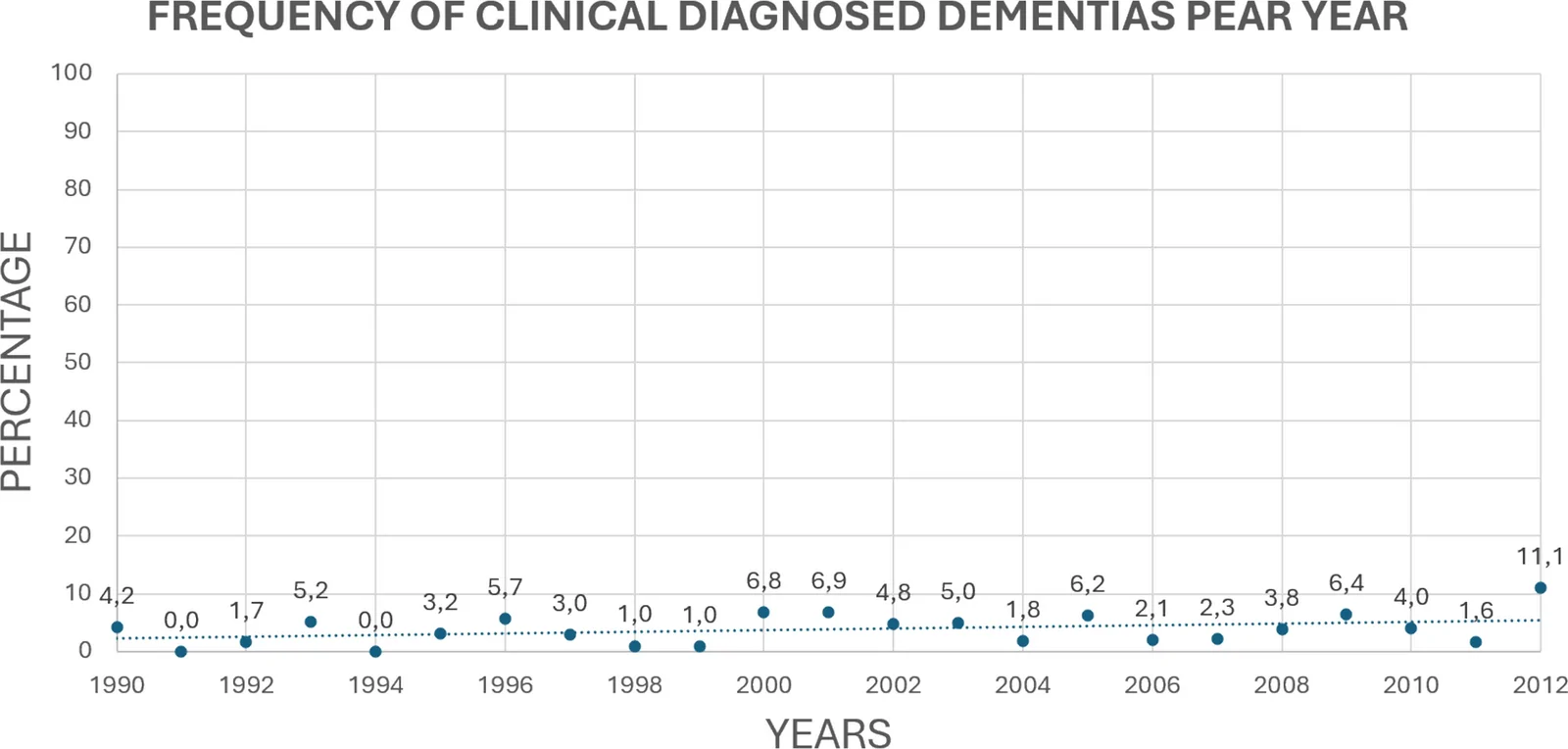
illustrates the annual prevalence of clinical diagnosed dementias, expressed as a percentage, across the study period from 1990 to 2012. Each data point represents the proportion of individuals diagnosed with dementia in a given year, while the fitted linear trend line depicts the overall temporal pattern

Dementia was present in 3.6% of the deceased individuals. The annual variation in the percentage distribution of dementia is illustrated in Fig. 9. Using a logistic regression model, no significant change was observed in the prevalence of dementia over the years.

### Histopathologically characterized dementia brain samples

Comprehensive neuropathological assessment revealed Alzheimer’s disease-related pathology in 38 brain samples and diffuse Lewy body disease pathology in 2 cases. Alzheimer’s disease cases were staged according to the Braak and Braak classification system, with 11 cases classified as Braak stages I–II, 21 cases as Braak stages III–IV, and 6 cases as Braak stages V–VI.

#### Parkinsonism

**Fig. 10:**
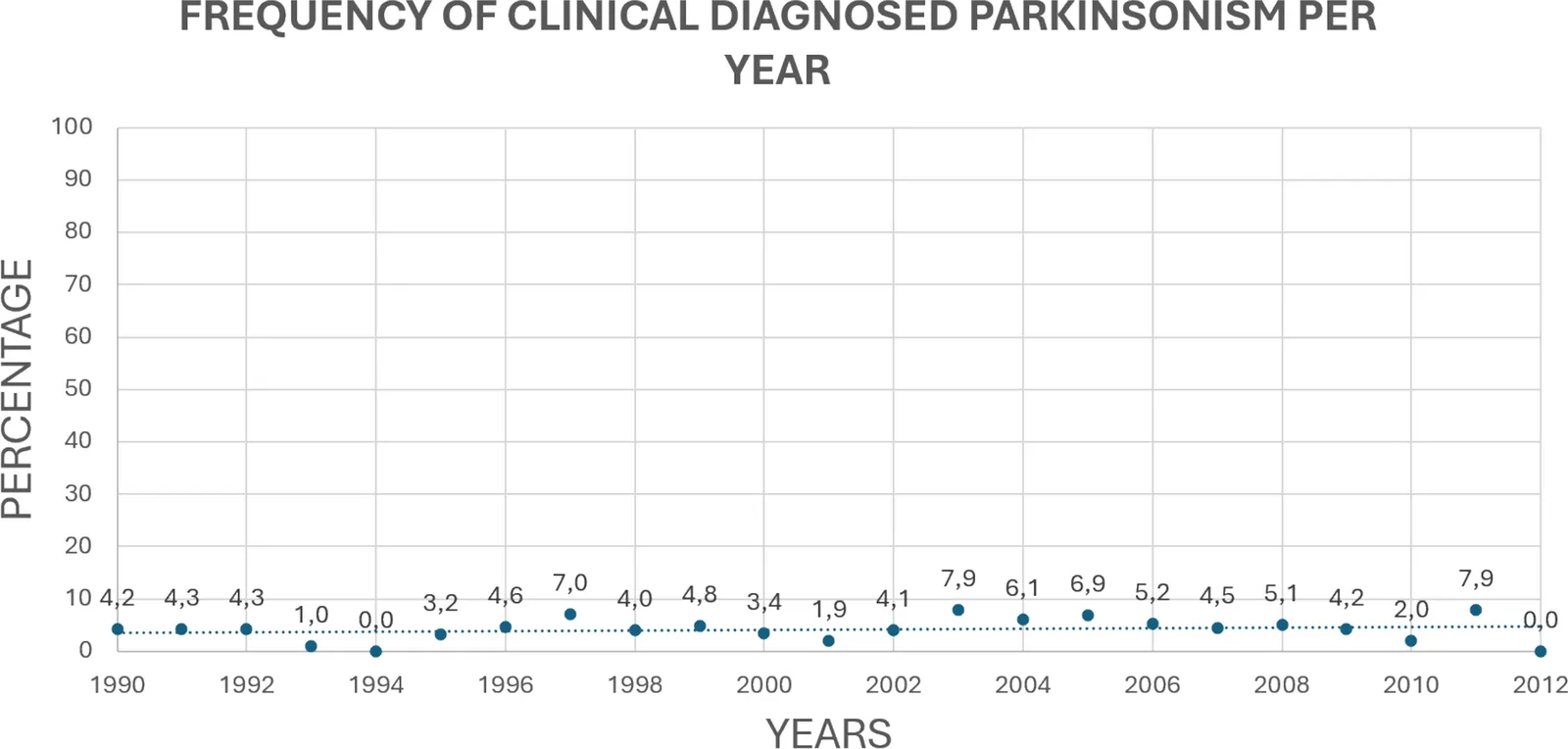
illustrates the annual prevalence of clinical diagnosed parkinsonism, expressed as a percentage, across the study period from 1990 to 2012. Each data point represents the proportion of individuals diagnosed with parkinsonism in a given year, while the fitted linear trend line depicts the overall temporal pattern

Parkinsonism was present in 4.5% of the deceased individuals. The annual variation in the percentage distribution of parkinsonism is illustrated in Fig. 10. Using a logistic regression model, no significant change was observed in the prevalence of parkinsonism over the years.

#### Psychiatric disorders

**Fig. 11:**
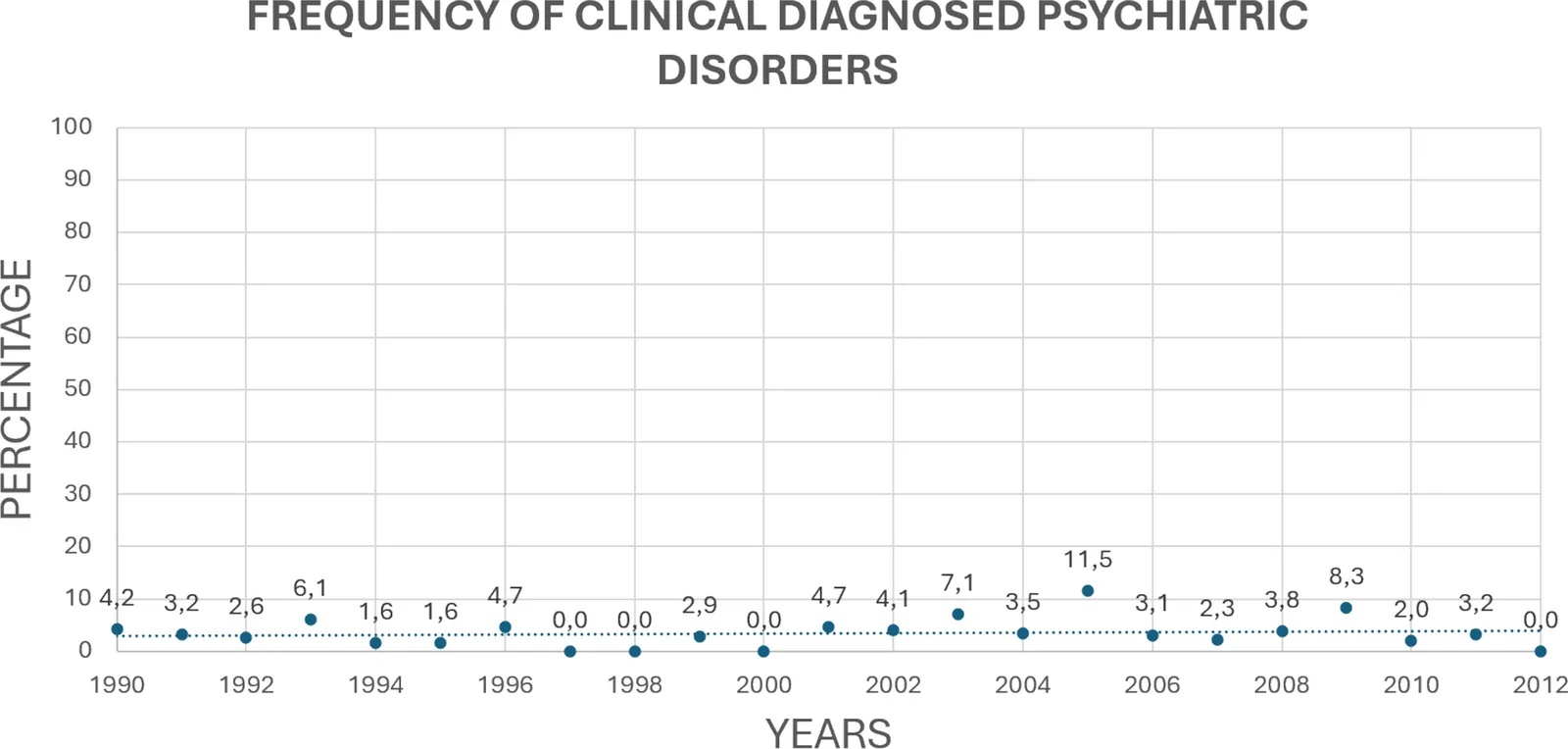
illustrates the annual prevalence of clinical diagnosed psychiatric disorders, expressed as a percentage, across the study period from 1990 to 2012. Each data point represents the proportion of individuals diagnosed with psychiatric disorders in a given year, while the fitted linear trend line depicts the overall temporal pattern

Psychiatric disorders were present in 3.8% of the deceased individuals. The annual variation in the percentage distribution of psychiatric disorders is illustrated in Fig. 11. Using a logistic regression model, no significant change was observed in the prevalence of psychiatric disorders over the years. Psychiatric disorders include bipolar depression, major and minor depression, anxiety disorder, schizophrenia, paranoid psychosis, hallucinations.

#### Distribution of causes of death

**Fig. 12:**
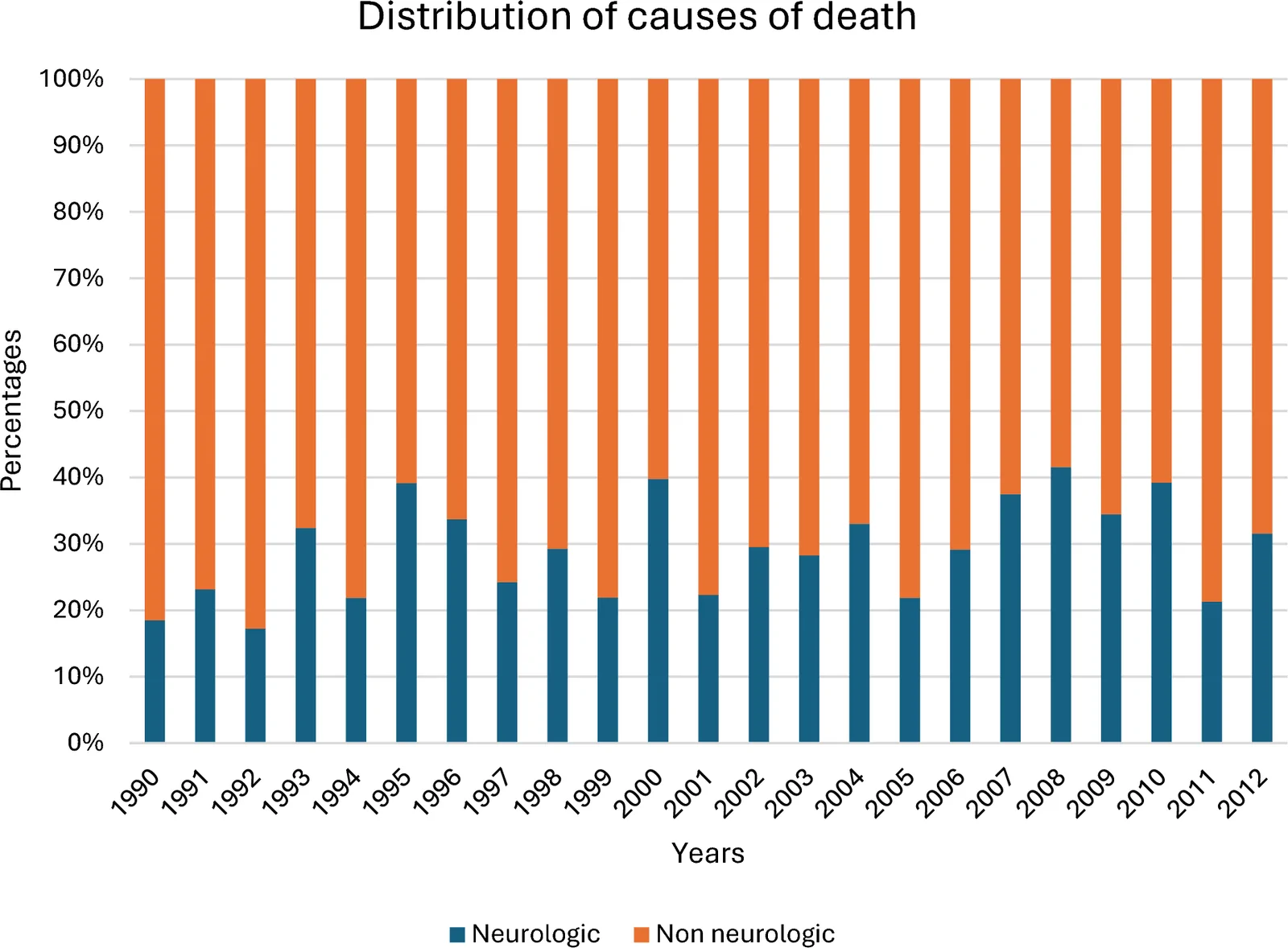
Annual variation in the percentage distribution of cause of death. Vertical axis indicates the percentages: death due to neurological condition is blue, while death due to non-neurological conditions is orange. The horizontal axis shows the years

Based on autopsy data 28.1% of patients die primarily due to neurological disorder, and 71.9% of patients die due to non-neurological conditions (e.g. pulmonary embolism, pneumonia, acute myocardial infarction, cardial failure, gastrointestinal haemorrhages). Distribution of causes of death is shown in Fig. 12.

#### Peripheral nervous system samples

**Fig. 13:**
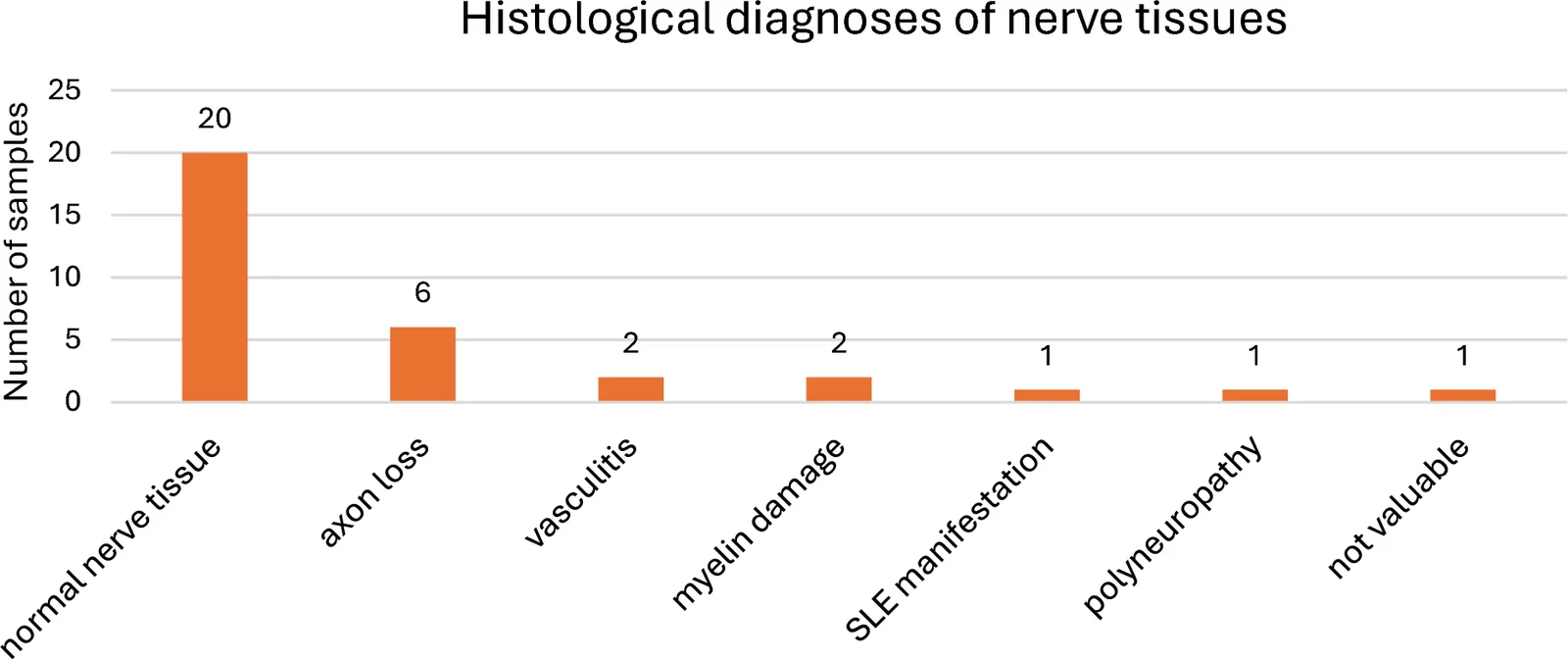
Distribution of histological diagnoses of nerve tissues

**Fig. 14:**
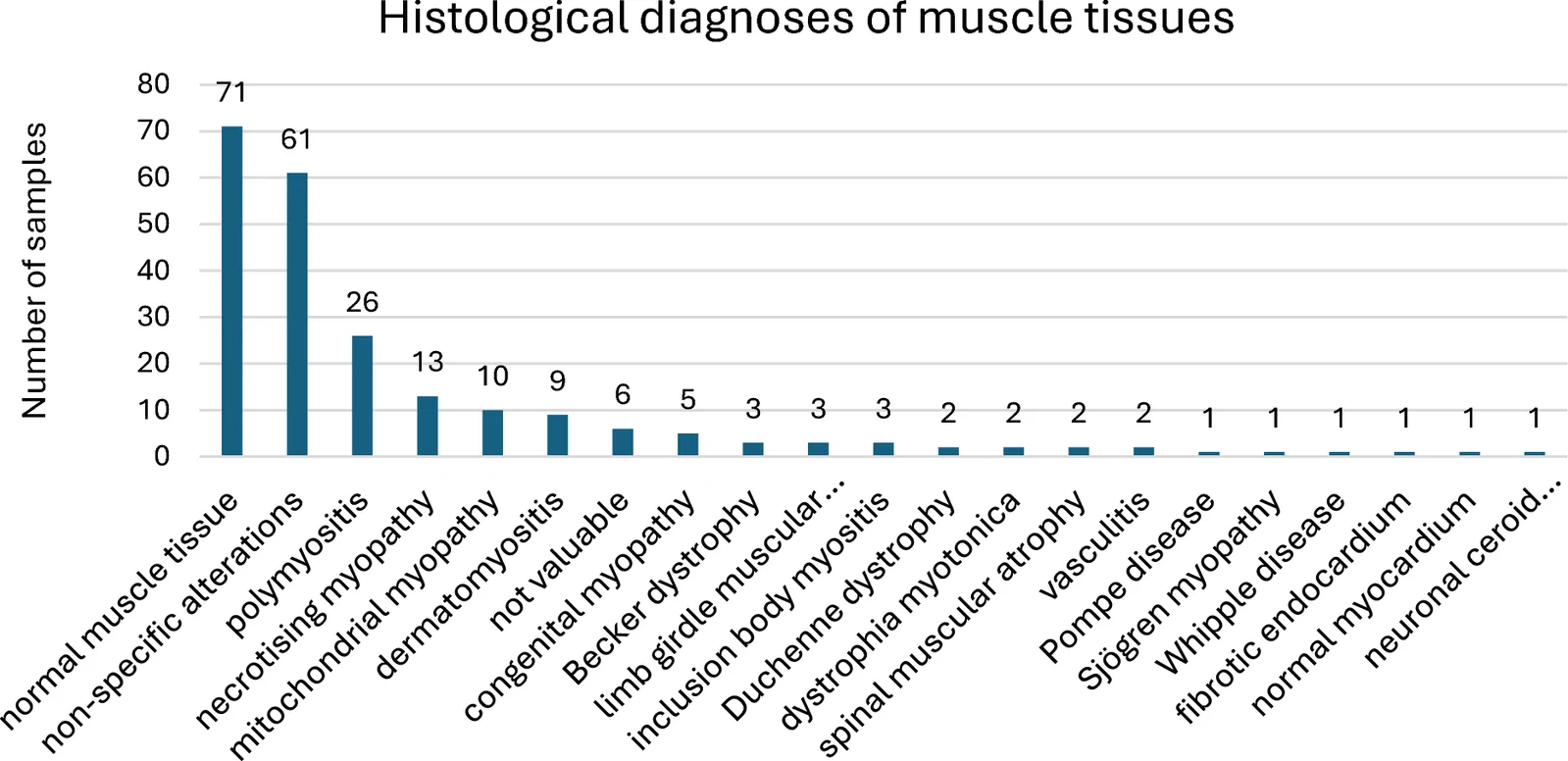
Distribution of histological diagnoses of muscle tissues

We have a total of 257 frozen peripheral nervous system tissue samples collected between 2014 and 2018, including 33 nerve biopsies and 224 muscle biopsies. All nerve biopsy samples are derived from the sural nerve. Muscle biopsy samples originate from the deltoid, quadriceps femoris, tibialis anterior, or gastrocnemius muscles. Of all samples, 39.7% (n=102) were obtained from male donors and 60.3% (n=155) from female donors. Among the nerve samples, 42.1% (n=14) were from males and 57.9% (n=19) from females. For the muscle samples, 39.3% (n=88) were from males and 60.7% (n=136) from females. Distribution of histological diagnoses of nerve tissues is shown in Fig. 13. Distribution of histological diagnoses of muscle tissues is shown in Fig. 14.

## Discussion

In this study, we presented the establishment of a new nervous system tissue bank in Hungary, along with statistical inferences drawn from the data accumulated during its development, which reflect the patient care profile of the Department of Neurology in Debrecen over a period of 22 years. As emphasized in the introduction, human tissue samples stored in the past represent a legacy: thanks to modern processing techniques, these samples serve as a valuable foundation for research aimed at uncovering pathomechanisms. The tissue bank of the University of Debrecen is unique in that it contains samples from patients who were previously treated at the Department of Neurology and later passed away, as well as nerve and muscle biopsy specimens from patients presumed to have neuromuscular diseases. The aim of our work was to review the material collected over the years and to link it with available clinical, pathological, and brain autopsy data. No new cases are being added to our tissue bank; our goal is to "deplete" the collection by releasing every single sample to researchers conducting the types of studies described in the introduction—to those who conduct research on human tissues. We have created a website to promote our tissue bank, which is accessible at www.udtissuebank.com. Interested researchers may request access to the detailed database; access is granted after we have received a brief summary from the research group outlining their previous work and the planned research involving our samples. All potential collaborations are strictly non-profit and can only proceed following the establishment of a research agreement with the University of Debrecen. Our database is anonymized, and no information contained therein allows for the identification of deceased individuals. A sample case from the searchable table developed for researchers is presented in Table 2. Upon request for access, all cases in our collection can be made available in this format. In addition, researchers may submit further specific requests, such as laboratory values at admission, during treatment, or details regarding pharmacological therapies administered throughout the clinical course.

**Table 2:** Sample case from the searchable table developed for researchers

General and submission data	
Lab ID	92/11
Sex	Female
Age at death	77
Days to death	2
Main indication of submission	ischemic stroke
Other information	basilar artery occlusion, ia. thrombolysis
Base diseases	
Hypertonia	1
Diabetes mellitus	1
Cancer	0
Psychiatric	0
Dementia	0
Parkinsonism	0
Other base diaseases/previous operations	
Cholecystectomy	
Struma opration	
Atrial fibrillation	
Chronic cardial failure	
General pathology	
Hypertonia related changes	
Myocardial fibrosis	
COPD	
Cor pulmonale	
Pneumonia	
Cause of death: pneumonia	
**Neuropathology**	
Post-mortem time: <24 h	
bilateral PCA territory ischaemic stroke with haemorrhagic transformation	
Alzheimer TAU pathology (Braak II stage)	
Available formalin brain	1
Available FFPE blocks	0

To the best of our knowledge, our tissue bank is one of the largest in Hungary and is remarkable even at European level in terms of the number of cases it contains. In Hungary, another tissue bank working with human central nervous system samples is located at Semmelweis University, established under the leadership of Professor Miklós Palkovits. This facility employs a unique processing technique known as the “Palkovits punch technique,” which involves microdissection of tissue blocks ranging from a few millimeters to 1–2 cm in size, stored by deep freezing. According to a report from a few years ago, tissue samples had been stored from approximately 280 brains, and this number has likely increased since then. (2016).

During the analysis of brain samples collected between 1990 and 2012, we extracted a range of data reflecting key indicators and the quality of hospital care during that period. These data were also included and analyzed as part of our study. In total, we processed 2425 cases. The number of stored samples remained consistently high throughout the 1990s and early 2000s, resulting in a substantial collection of brain samples from this period. However, starting in 2007, a marked and irreversible decline in autopsy numbers began— a trend that aligns with international patterns, as autopsy rates have been decreasing globally. (Hudák et al. 2022) Except for a few years, the proportion of male decedents was higher than that of female decedents in most years. This finding is consistent with data reported in the literature, which indicate higher hospital mortality rates among men—particularly in older age groups—compared to women. Key contributing factors include smoking, cardiovascular disease, hormonal and genetic differences, as well as psychosocial habits and behavioural patterns. (Wu et al. 2021) Our study demonstrated a gradual increase in the age of deceased individuals over the years. This trend may be related to findings reported by Wéber et al., who observed a rise in life expectancy at birth across 28 European countries during the 1995–2019 period. The primary factors underlying this increase likely include the earlier detection and timely treatment of chronic diseases, particularly cardiovascular conditions and cancer. (Wéber et al. 2023) In the population examined in our study, the number of hospital days prior to death gradually decreased over time. This trend may be associated with an overall decline in mortality rates during the observed period, as indicated by the decreasing number of autopsies performed. The likely explanation is that medical diagnostics and treatment became more effective, resulting in fewer deaths among patients whose conditions could be managed successfully. Consequently, those who did pass away were predominantly individuals with more severe conditions for which no effective therapy was available, leading to relatively rapid disease progression and death. The distribution of admission diagnoses in our cohort largely corresponds to those reported in the Global Burden of Diseases, Injuries, and Risk Factors (GBD) Study. Notably, cerebrovascular diseases were the most prevalent, followed by admissions due to brain tumors and epilepsy. Since the most common neurological disorders identified in the GBD Study—tension-type headache and migraine—typically do not require inpatient admission, they are not represented in our study population. Additionally, dementias appear to be underdiagnosed within our cohort. (GBD 2015 Neurological Disorders Collaborator Group 2017) Over time, the prevalence of diabetes mellitus and hypertension as underlying conditions increased significantly in our population. This trend diverges from patterns observed in Europe; for example, a Spanish study reported a decline in the incidence of both diabetes mellitus and hypertension during the twenty-first century, attributing this improvement to effective national preventive programs. These findings suggest that preventive measures in Hungary may require further enhancement. (Bennett et al. 2023) The prevalence of neoplastic diseases remained relatively stable over the years, with no significant changes observed. The most common non-neurological tumor types were lung, colorectal, and breast cancers, consistent with international trends. (Bray et al. 2024) The incidence of brain tumors in our study population was markedly higher than reported in the literature, which is unsurprising given that our cases comprised patients who died in the neurology department, where such conditions are concentrated. No significant changes were observed over the years in the prevalence of dementia, parkinsonism, and psychiatric underlying conditions within the study population. An important question is whether patients treated in neurology departments primarily die as a result of their neurological condition or due to extraneural causes. Our study’s findings indicate that the vast majority of these patients succumb to extraneural causes, most commonly thromboembolic complications, pneumonia, cardiac arrhythmias, or gastrointestinal bleeding. These extraneural causes primarily arise as a consequence of immobilization or functional impairment resulting from the underlying neurological disease. This underscores the critical importance of proactively addressing the prevention of such extraneural causes of death during patient care, as well as ensuring their prompt diagnosis and treatment should they occur. Few studies have examined this issue; however, available data from the international literature report similar findings. (Kompoliti et al. 2017).

The findings presented in our study emphasize the importance of performing autopsies, as the information gained is crucial for improving the quality of hospital care and for obtaining human tissue samples necessary for scientific research. Should our work have raised your interest and you require human tissue samples for your study, please visit www.udtissuebank.com.

## „Hic mortui vivos docent”

### Limitations

This study has several limitations. First, due to its retrospective design, the analysis was restricted to the availability and quality of historical clinical and neuropathological data, which were not collected according to standardized protocols throughout the entire study period. In particular, before 2010, neuropathological examinations were primarily limited to macroscopically affected regions, precluding comprehensive assessment of neurodegenerative pathology. Second, the study population is subject to selection bias, as the majority of cases were patients admitted with acute, life-threatening neurological conditions, most prominently cerebrovascular diseases. Consequently, neurocognitive disorders are likely underrepresented in our cohort, both due to the clinical focus on acute conditions and the limited feasibility of detailed cognitive assessment during terminal hospitalizations. This is further supported by the relatively low proportion of clinically diagnosed dementia cases compared to epidemiological data. Finally, changes in autopsy rates and tissue sampling practices over time may also have influenced the observed temporal trends.

## Data Availability

The datasets generated and/or analysed during the current study are not publicly available due it contains sensitive patient information, but are available from the corresponding author on reasonable request.
